# Biophysical nanocharacterization of liver sinusoidal endothelial cells through atomic force microscopy

**DOI:** 10.1007/s12551-020-00699-0

**Published:** 2020-05-18

**Authors:** Bartlomiej Zapotoczny, Filip Braet, Eddie Wisse, Malgorzata Lekka, Marek Szymonski

**Affiliations:** 1 Institute of Nuclear Physics, Polish Academy of Sciences, 31-342, Krakow, Poland; 2 Faculty of Medicine and Health, School of Medical Sciences (Discipline of Anatomy and Histology), The University of Sydney, Sydney, NSW 2006 Australia; 3 Australian Centre for Microscopy & Microanalysis, The University of Sydney, Sydney, NSW 2006 Australia; 4 Charles Perkins Centre (Cellular Imaging Facility), The University of Sydney, Sydney, NSW 2006 Australia; 5 Maastricht Multimodal Molecular Imaging Institute, Division of Nanoscopy, University of Maastricht, Maastricht, Netherlands; 6 Research Centre for Nanometer-Scale Science and Advanced Materials, NANOSAM, Faculty of Physics, Astronomy and Advanced Computer Science, Jagiellonian University, Krakow, Poland

**Keywords:** Cell Nanoscopy, Cytoskeleton, Elasticity, Fenestrae/fenestrations, Force mapping, Live cell imaging

## Abstract

The structural-functional hallmark of the liver sinusoidal endothelium is the presence of fenestrae grouped in sieve plates. Fenestrae are open membrane bound pores supported by a (sub)membranous cytoskeletal lattice. Changes in number and diameter of fenestrae alter bidirectional transport between the sinusoidal blood and the hepatocytes. Their physiological relevance has been shown in different liver disease models. Although the structural organization of fenestrae has been well documented using different electron microscopy approaches, the dynamic nature of those pores remained an enigma until the recent developments in the research field of four dimensional (4-D) AFM. In this contribution we highlight how AFM as a biophysical nanocharacterization tool enhanced our understanding in the dynamic behaviour of liver sinusoidal endothelial fenestrae. Different AFM probing approaches, including spectroscopy, enabled mapping of topography and nanomechanical properties at unprecedented resolution under live cell imaging conditions. This dynamic biophysical characterization approach provided us with novel information on the ‘short’ life-span, formation, disappearance and closure of hepatic fenestrae. These observations are briefly reviewed against the existing literature.

**Electronic supplementary material:**

The online version of this article (10.1007/s12551-020-00699-0) contains supplementary material, which is available to authorized users.

## Introduction

The microvascular endothelium constitutes a monolayer of cells lining the inner surface of blood vessels. As such, this layer of endothelial cells forms a structural-functional barrier between the circulating blood and surrounding tissue. Being continuously exposed to flowing blood, integrating physical and chemical signals from the surrounding tissues, the endothelium is involved in various physiological and pathological processes (Chiu and Chien, 2011; Cahill and Redmond 2016; Krüger-Genge et al. 2019). With the presence of a wide repertoire of mechanosensors and receptors, the endothelium regulates velocity of blood flow and bidirectional transport from and into the blood stream (Azuma et al. 2000; Chatterjee 2018; Yazdani et al. 2019). The transportation of substances occurs both via diffusion and by specialized receptor-based transport processes (Yazdani et al. 2019). Dependent on the location in the organism, the morphology and function of endothelial cells shows a remarkable degree of complexity (Risau 1995; Satchell and Braet 2009). For example, endothelium in the brain constitutes a strict blood brain barrier, allowing for unique receptor-based selective transport of nutrients. Endothelial cells in the aorta are large and stiff, with a prominent cytoskeleton to resist the high pressure of flowing blood. In contrast, liver sinusoidal endothelial cells (LSECs) are the most permeable type of endothelium. Six to eight percent of their perinuclear zone is covered with fenestrae (or fenestrations) which are open transcellular pores without the presence of a diaphragm. Noteworthy, besides fenestrae, LSECs have an abundant presence of endocytic vesicles embedded within the non-fenestrated cytoplasm (Wisse 1970). Fenestrae are typically gathered in groups of ~ 10–100 pores, called sieve plates and they lack a basal lamina. The size of fenestrations is in the range of 50–300 nm. This allows them to efficiently and freely transport solutes and particles through the liver sinusoidal endothelium barrier (Wisse et al. 1985). LSEC sieve plates are as such the structural gatekeepers tightly controlling transport in a size-dependent manner, excluding larger matter such as cells or chylomicrons (Fraser et al. 1995). Finally, LSEC fenestrae have been shown to be highly dynamic in nature with respect to their size and number resulting in a decreased or increased sieving function (Braet and Wisse 2002).

Generally speaking, the endothelium stands central in tissue and organ homeostasis, and as a consequence has remarkable diagnostic and therapeutic potential. Endothelial cells actively participate in homeostasis. They regulate vascular tone via the production of nitric oxide (NO), endothelin and prostaglandins (Gryglewski et al. 1995; Vanhoutte et al. 2016). They have antithrombotic properties and are involved in atherogenesis (Moncada et al. 1977). There is a fine balance between expressing and/or secreting surface receptors and soluble mediators, and hence maintaining proper endothelial cell phenotype. Furthermore, the crosstalk between the surrounding tissue and endothelial cells influences their morphology (Maslak et al. 2015), organization of cytoskeleton and mechanical properties (Pratt et al. 1984). In particular, changes in the organization of LSEC-cytoskeleton were shown to alter the number and size of fenestrae, and thus the filtration efficiency of LSECs (Steffan et al. 1987). Different elements of the cytoskeleton were reported to affect the porosity of LSECs, like actin, tubulin, myosin and spectrin (Steffan et al. 1987; Braet et al. 1996a; Gatmaitan et al. 1996; Zapotoczny et al. 2019a). Among others, the actin cytoskeleton has been studied widely in the last decades (Braet et al. 1998b; Braet and Wisse 2002; Hunt et al. 2019). Modifications in actin organization, especially using a variety of actin-targeted agents derived from marine natural sponges, provided data on fenestrae-forming centres (FFC), defenestration centres (DFC) and different organization of fenestrae in sieve plates (Braet et al. 1998b, 2003; Braet 2004). The question arises, whether those modifications in actin are responsible for changes in the nanomechanical behaviour of LSECs and vice versa, and whether the alterations in nanomechanics are reflected in the porosity of endothelium. Those questions sparked our interest to gather a better insight on LSEC dynamics utilizing scanning probe microscopy approaches (Braet et al. 1998b; Zapotoczny et al. 2017c) and in particular to assess the elastic properties (i.e. elasticity versus stiffness) of LSEC, including the different structural organizations of fenestrae and their associated cytoskeleton. This thematic paper aims at providing an overview of the recent advances and insights gathered on the nanomechanical behaviour of LSEC-fenestrae under different experimental conditions. In particular, various biophysical aspects will be used that are relevant to visualize (sub)membranous changes of the LSEC cytoskeleton in particular how these changes affect the porosity. For interested readers, a few other recent reviews are recommended focussing on different aspects of fenestrae and LSECs (Maslak et al. 2015; Sørensen et al. 2015; Braet et al. 2018; Cogger et al. 2020; Sørensen and Smedsrød 2020).

## Atomic force microscopy as a biophysical nanotool for LSEC characterization

Atomic force microscopy (AFM) is a member of the scanning probe microscopies allowing the investigation of the biophysical properties of cells in vitro (Radmacher et al. 1992). During measurements, the tip of the cantilever is scanning the surface of a sample. The loading force exerted by the tip causes the deflection of the cantilever which corresponds to the mechanical properties of investigated object. In the years since the development of the AFM, it has become a versatile tool used to quantify the properties of biological objects, including the surfaces of cells in vitro (Dufrêne et al. 2017). Several approaches were proposed to use AFM to measure elasticity, organization of the cytoskeleton, cell’s size and height, or even the organization of membrane proteins (Sokolov et al. 2007; Targosz-Korecka et al. 2017). The variety of deviations from physiological conditions of endothelial cells has been already shown to be somehow reflected in the biophysical properties of investigated cells. For example, during inflammation, hyperglycaemia or hypertension, changes in both morphology and nanomechanical properties of endothelial cells have been documented in detail (Oberleithner et al. 2011; Szczygiel et al. 2012; Zhang et al. 2015; Malek-Zietek et al. 2017). In a recent review, we described the progress made to date on the various types of liver sinusoidal cells and hepatocytes utilizing AFM, including LSECs (Braet et al. 2018). Briefly, reviewing the AFM-liver literature disclosed that this probing technique allows to monitor the biophysical properties of liver cells under different experimental conditions at unprecedented resolutions, and this in multiple dimensions (X,Y,Z&t, referred here as 4-D). More recently, taking advantage of our 4-D AFM approach (Zapotoczny et al. 2017c), we employed the AFM to evaluate the progress of experimentally induced fatty liver disease in mice (Kus et al. 2019).

The presence of fenestrae and sieve plates is a hallmark to determine the physiological versus pathophysiological states of LSECs. Variations in size and diameter can be used as morphological indicators for their health status. The nanometre-size transcellular pores were reported to respond to a variety of external stimuli becoming a great marker for the functional responsiveness of LSECs. Interestingly, there are only a few reports available, detailing the elastic properties of LSECs (Braet et al. 1998b; Kus et al. 2019; Zapotoczny et al. 2019a). More than two decades ago, a first attempt was made to depict fenestrae in living LSECs. Unfortunately, due to AFM hardware limitations and the absence of ultrasoft cantilevers at that time, fenestrae could not be captured with this nanoscale imaging approach (Braet et al. 1997, 1998a). Moreover, during these studies, the authors determined that LSECs possess extraordinary soft properties (i.e. ~ 2 kPa).

Recently, we documented in detail the nanomechanical properties of this type of fenestrated sinusoidal endothelium exposed to various experimental conditions under live cell imaging conditions (Zapotoczny et al. 2017a, 2019a; Rusaczonek et al. 2019). Furthermore, significant progress was made in the detailed analysis on the organization of the cytoskeleton associated with fenestrae and sieve plates. Developments in AFM hard- and software over the past 10 years (e.g. fast data acquisition, improved piezo elements performance, accurate temperature control, etc.) allowed us as first to have a glimpse on the highly dynamic nature of fenestrae. The proceeding sections will highlight how the latest advances in AFM imaging and associated image analysis contributed to a better understanding in the origin, dynamics and structural arrangement of fenestrae and sieve plates, including the various (sub)membranous structures associated with the fenestrated cytoplasm. We propose herein a practical approach of coupling porosity and elasticity as determinants in the ‘morphomechanics’ of LSECs. We indicate that the elasticity together with the numerical dynamics of fenestrae could be considered strong morphological markers to determine the physiological and pathological states of LSECs.

### Recent advances on AFM measurements in live and fixed LSECs

**Fig. 1:**
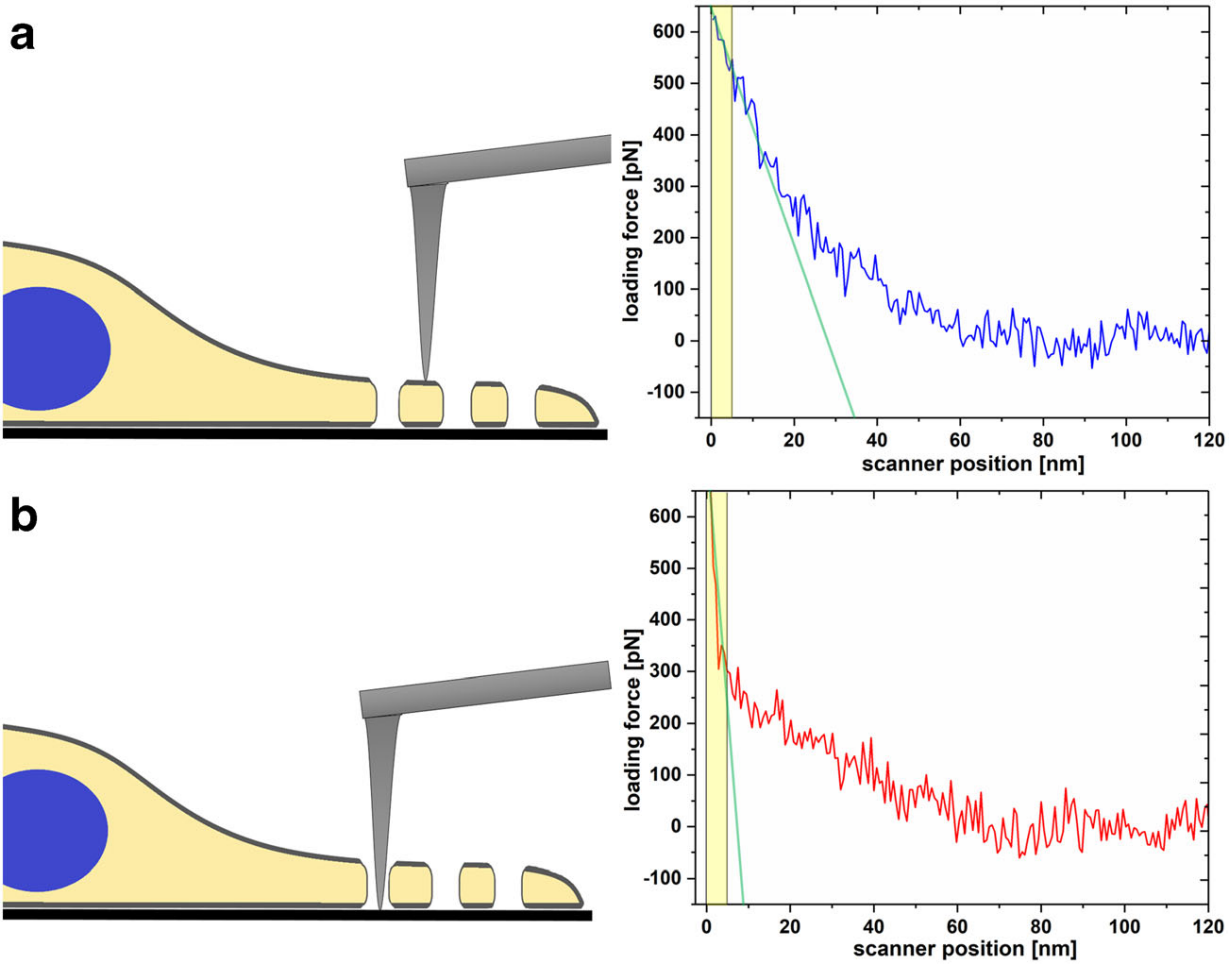
The differences in the slope of the FD curves are used to create clear contrast in the AFM image of presenting stiffness distribution. (a) Corresponds to FD curve collected on a soft cell, where the gentle increase of the loading force and simultaneous change in scanner position (corresponding to the indentation) is observed. (b) Corresponds to FD curve collected on a stiff glass slide within the open fenestra, where the sharp increase of the loading force and negligible indentation is observed (prepared based on Fig. 4 from (Zapotoczny et al. 2017c))

In 2017, we showed that the latest advances in AFM can be successfully applied to imaging both fixed and live LSECs (Zapotoczny et al. 2017c, b, a). The methodology based on the collection of force-distance (FD) curves allowed for minimizing lateral forces, thus, enabled detailed investigations of fragile samples. In particular, quantitative imaging mode (QI) was applied in previous reports (Chopinet et al. 2013; Zapotoczny et al. 2017c). During the QI imaging, in each pixel point, an independent FD curve is recorded, allowing the detailed post-processing of obtained data. For instance, the topography of cell surfaces can be reconstructed for a selected loading force, starting from the contact point up to the maximal loading force used in the measurement. Additionally, by calculating stiffness for the largest loading force of each FD curve, it is possible to obtain a clear contrast between the stiff glass slide and the soft cell (Fig. 1)(Zapotoczny et al. 2017c).Such tremendous contrast is achieved because of the fact that fenestrae are open transcellular pores, without a membranous diaphragm as in some other endothelia (Risau 1995). When the tip apex touches the glass underlying the fenestra, with increasing the loading force, no significant indentation is observed. In contrast, when FD curves are collected on a soft cell surface, subsequent deformation (indentation) of the cell and an increase of the loading force are recorded. Calculations of the stiffness (defined as the slope of the FD curve) in the narrow part of FD curves—corresponding to the large loading force (yellow region in Fig. 1) —lead to highly contrasted images. Such approach was reported to be particularly useful, when large images of the whole cells were collected. Point-to-point resolution is often set above 100 nm. It allowed for fast (5–20 min/frame) screening of the porosity of entire LSECs. Such resolution is unfortunately too low, hampering detailed calculations of the number and size of individual fenestrae. Selecting high spatial resolution during large image acquisition resulted in slow imaging speed (slower than dynamics of cell natural processes) resulting in deformed images. Therefore, when high-resolution imaging (i.e. point-to-point resolution below 40 nm) was necessary, an area of several μm^2^ was typically selected.

Better contrast showing surface details can be obtained from a reconstruction of cell topography instead of calculation of the stiffness. By applying low loading force in the order of 200–300 pN, images of cell topography clearly show fenestrae. Low loading force allowed for minimizing the alterations in the topography of measured cells being a result of deformation caused by the pressure exerted by the tip on the (sub)membranous cytoskeletal structures. According to a variety of reports, the indentation resulted from a loading force of 200–300 pN corresponds to the cell’s cortex layer (Fels et al. 2012; Szymonski et al. 2015). Larger loading forces (> 500 pN) combine the mechanical response of cortex and the deeper parts of cell, including the nuclear envelope. Similarly in LSECs, loading forces set within the above mentioned range show actin stress fibres and fenestrae-associated cytoskeleton rings (FACRs) (Zapotoczny et al. 2017c).

FD-based imaging techniques provide additional advantages as several types of artefacts, typical for imaging in contact mode AFM can be avoided. For example, streaks in the direction of scanning are often observed (Braet et al. 1997). They highly probably result from the lateral deformation of soft biological structures induced by scanning direction (Zapotoczny et al. 2017b). It was reported that QI allows for the application of even sharpened cantilevers (tip apex radius of 2–12 nm) with a great spatial resolution on fixed LSECs (Zapotoczny et al. 2017b).

The main limitation of force imaging is the time required to acquire an individual FD curve. As mentioned before, an independent FD curve is recorded in each pixel point of the image. Therefore, high spatial resolution requires dense matrix of FD curves, which means selecting areas of only several μm^2^ or increasing time required for the acquisition of single frame for larger areas. Thus, presented up to date animations are a compromise between the resolution and speed of imaging. Both parameters have to be adapted to the biological processes under study. For example, images of a spreading LSEC during the first hours after isolation were performed using a large area (15 × 15 μm^2^) with 15 min per frame (Zapotoczny et al. 2019b). In contrast, the rapid effect of cytochalasin B on the formation of new fenestrae and FFC was performed using a small area (5 × 5 μm^2^) at low resolution (100 × 100 points), but was acquired with only 45 s of time per frame (Zapotoczny et al. 2017c).

The second approach to analyse the effect of different agents on LSECs morphology started with the fixation of LSECs at predefined time points. Such approach has been applied several times before using mainly electron microscopy (Braet et al. 2003; DeLeve et al. 2004; Xie et al. 2012). Investigation of fixed cells has many advantages. It allows for the comparison of groups of cells, both treated and untreated (control). The increase in LSEC stiffness under fixed conditions also inherently benefits the final resolution that can be achieved. This approach permits detailed and statistical quantification of the effects of different factors and agents on LSECs. Comparative analysis on cell characterization requires the availability of descriptive parameters, such as structural and/or functional markers that can be quantified. Quantification of the parameters describing LSECs (both isolated and in situ) were done more than five decades ago and covered the following items: the porosity, i.e. the percentage of LSEC surface covered with fenestrae, the fenestrae number per μm^2^, the fenestrae diameter (often presented as the distribution of fenestrae diameters) and presence of gaps—openings larger than 400 nm in diameter (Wisse 1970; Steffan et al. 1987). Since then, those parameters have been widely presented in the literature describing fixed LSECs with mainly electron microscopy-based methods (for reviews, see (Braet and Wisse 2002; Cogger et al. 2013)). Recently, a novel optical nanoscopy, as stimulated emission depletion microscopy (STED), 3D structured illumination microscopy (3D-SIM) or direct stochastic optical reconstruction microscopy (dSTORM)(Mönkemöller et al. 2015; Di Martino et al. 2018; Hunt et al. 2018; Øie et al. 2018), also resolved fenestrae in isolated LSECs. The comparison between different groups of drug-treated LSECs has been recently reported (Hunt et al. 2018).

**Fig. 2:**
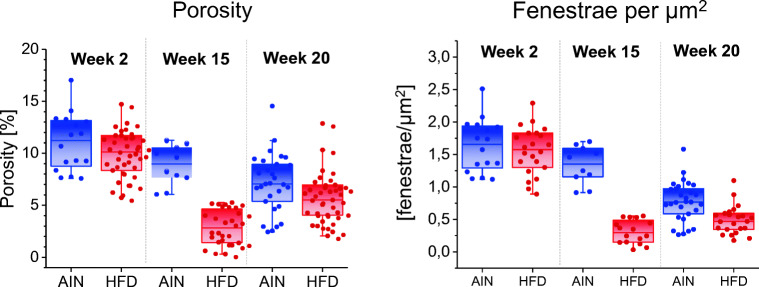
Morphological changes in primary LSECs isolated from C57Bl/6 mice during non-alcoholic fatty liver disease (NAFLD) progression evaluated by AFM technique. The results of LSECs porosity and number of fenestrae per μm^2^ were compared for samples taken from mice being on control (blue, AIN) and high-fat (red, HFD) diets

Having the methodology set independently for scanning electron microscopy (SEM) and AFM, experiments were performed that allowed full comparison of the results within the same animals. Our results presented with AFM were in good agreement with those obtained using electron microscopy (EM)(Kus et al. 2019). The most prominent difference between these techniques was reflected in the diameter of fenestrae (discussed further in the section ‘fenestrae diameters’). Kus et al. compared the phenotype of LSECs isolated from mice being on both high fat and on control diets. The study provided, among others, data on alterations in porosity of LSECs with progression of non-alcoholic fatty liver disease (Kus et al. 2019). Both techniques provided similar results about the porosity of LSECs. We extended previously presented data (Kus et al. 2019) by adding new experiments. AFM results demonstrated that there is an age-related constant decrease in the porosity of isolated LSECs (Fig. 2).

It was previously reported that the porosity of LSECs decreases with age; e.g. in experiments performed with perfused liver in young versus old rats (Simon-Santamaria et al. 2010); and inhuman liver sections (McLean et al. 2003). Our results indicate that the determination in the number of fenestrae is directly translatable from the in vivo to the in vitro experiment and vice versa. After LSEC isolation, the decreased porosity with age is also observed in culture. Moreover, this observation is the first, in which AFM reproduced a known biological effect observed in situ first, and later reproduced in vitro. This clearly underpins the future promise of AFM in research settings, including preclinical screening to evaluate LSEC response after different treatment regimes.

### Real-time monitoring of live LSEC-fenestrae

**Fig. 3:**
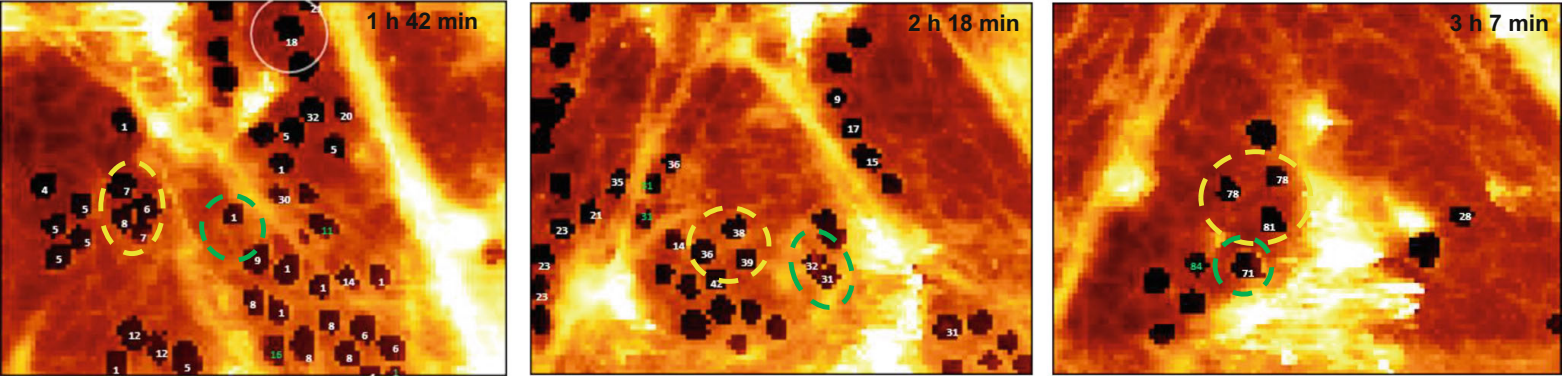
Selected area of LSEC measured using 4-D AFM after 24 h from seeding on a glass slide. Two groups of fenestrae divided with sieve-associated cytoskeleton ring were indicated using green and yellow dashed outlines. Overtime fenestrae became located within the same sieve plate and eventually they switched their positions (these images are selected from a 4 h long animation presented as Supplementary animation 5 elsewhere (Zapotoczny et al. 2019b))

As described above, the fixation of LSECs allows the detailed analysis of the morphology of LSECs in one selected time frame in X, Y and Z. Although this approach is very useful for certain experiments, it narrows the information obtained during the measurement. With the development of advanced 4-D (X,Y,Z&t) AFM approaches, LSEC quantification reached a new level. It allowed not only to quantify structural parameters such as porosity and/or fenestrae diameters in 3-D (X,Y&Z) but also permitted to track the dynamics of some parameters at a high temporal resolution (i.e. time)(Zapotoczny et al. 2017c, 2019b). In fact, AFM revealed that fenestrations are dynamic structures migrating through sieve plates and that their number and size are continuously changing in accordance with the reorganization of cellular cytoskeleton. AFM assessed the lifespan of fenestrae in LSECs cultured for more than 12 h, i.e. 75% of fenestrae remained open for less than 20 min and only 5% of fenestrae remained open for more than 1 h (Zapotoczny et al. 2019b). 4-D AFM is the only type of microscopy form available to date that was able to unambiguously disclose that fenestrae migrate within the sieve plate, independently on the whole cell migration process. Note that changing of their position can be also independent on sieve plates (Fig. 3).

In Fig. 3, groups of fenestrae of two independent sieve plates were monitored over time with 1-min period between two consecutive images (Fig. 3, green and yellow dashed outlines). During the first hour, they migrated and became grouped in the same sieve plate. Eventually, we could observe that those groups of fenestrae move towards each other and even switched their orientations relative to one another after 1 h and a half. This observation may indicate that the FACRs (Braet et al. 1995) are remarkably stable structures that also allow fenestrae to move freely and independently away for several micrometres.

### Fenestrae diameters

4-D AFM also revealed new information about diameter of fenestrae. We determined that individual fenestrae could increase their diameter up to 300% during their lifespan (Zapotoczny et al. 2019b). The observed changes in individual fenestrae diameters in live LSECs opens the debate about the genuine size of fenestrae as observed in SEM, TEM. In fact, it was presented that the process of fixation caused shrinking of fenestrae diameters (Braet et al. 1994; Gatmaitan et al. 1996). In summary, the mean size of fenestrae varies between live and fixed cells up to 30%, i.e. 180 nm ± 41 nm for live cells, ${143}_{-27}^{+48}$ nm for wet-fixed cells up to 130 ± 41 nm for fixed-dried cells (Steffan et al. 1987; Braet et al. 1996b; Zapotoczny et al. 2017b, 2019b). The shrinkage of fenestrae was also valid for the tissue as a whole (Wisse et al. 2010), and therefore the most accurate values of fenestrae diameters of ~ 140 nm seem to be obtained for TEM cryo-sections (Wisse et al. 1985; Snoeys et al. 2007).

### The eventual-ceasing fenestrated morphology

EM studies have shown that the number of fenestrae decreased with time and also that fenestrae vanished after 2–3 days in culture (Funyu et al. 2001; Sellaro et al. 2007; Xie et al. 2012). In the first hours after LSEC isolation, individual fenestrae and whole sieve plates were readily visible, when LSECs spread on a glass slide (Zapotoczny et al. 2019b). The high speed in the processes of fenestrae formation, disappearance and migration hampered tracking of individual fenestrae, and thus the accurate assessment on the lifespan of fenestrae (Supplementary animation 1). With culture time we observed less evidence of the continuing formation of new fenestrae. However, an animation performed after 48 h after isolation (Supplementary animation 2) indicates that fenestrae are still dynamic in nature but there is less new fenestrae formation, which resulted in a gradual decrease of the overall porosity in time. We believe that the process of defenestration (referred here as a loss of fenestrae) in vitro is ascribed to the overall reduced ability of LSECs to create new fenestrae overtime. Moreover, constant creation of new fenestrae de novo in cultured LSECs indicates that the total number of fenestrae is not directly preserved from the tissue in vivo. In fact, it was shown that the number of fenestrae of isolated LSECs was twofold lower than the number of fenestrae in tissue (Steffan et al. 1987). For future experimentation, it should be considered during the assessment of the porosity in cultured LSECs to fix the cells at certain time frames after cell isolation to collect additional insights on this intriguing matter.


ESM 1(MP4 29,203 kb)



ESM 2(MP4 8293 kb)


### Dysfunctional endothelium in vitro—quest for pharmacology in real-time

**Fig. 4:**
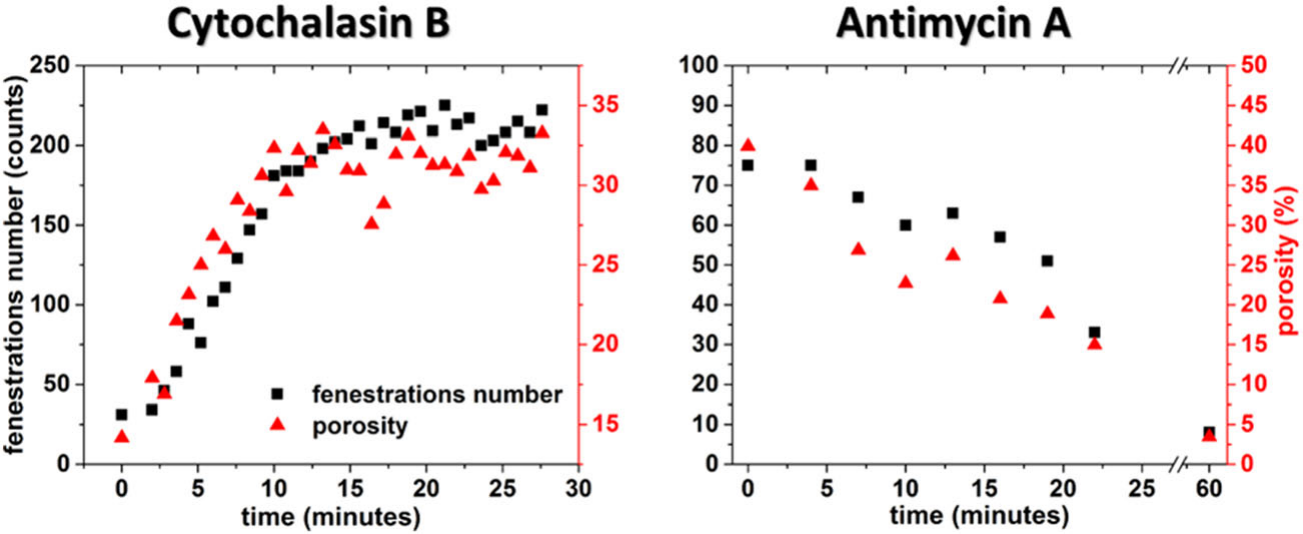
Time dependence of fenestration number and porosity of LSECs membrane after treatment with 10 μg/ml cytochalasin B and 1 μg/ml antimycin A (reprinted with permission from (Zapotoczny et al. 2017c))

The quest for the dynamic nature of fenestrae is not new. Steffan et al. showed that fenestrae could be induced under in vitro conditions when the actin cytoskeleton is disrupted (Steffan et al. 1987). Since then, different factors and disease models were investigated using EM of cells fixed after certain periods of treatment (which were summarized in Table 2 (Braet and Wisse 2002) and Table 2 (Cogger et al. 2013) of the corresponding reviews). With the AFM-based methodology outlined in this paper, one can study the different LSEC-cytoskeleton components which affect the number and/or size of fenestrae. Using 4-D AFM, the fenestrae responses to a certain drug formulation can be readily assessed. Our method allows adding an agent (drug or toxin) to the medium and continues measurements in the same region (Zapotoczny et al. 2017c, 2019a, b). This allowed us to observe the real-time response of the cell to an agent. For example, we showed that the effect of cytochalasin B was rapid and led to the reduction of cell height and to the formation of new sieve plates and fenestrae (Fig. 4).

As soon as after 15 min, the effect of the cytoskeletal-altering drug saturated. On the other hand, antimycin A caused continuous reduction of number and size of fenestrae leading to the almost complete loss of fenestrae after 1-hour treatment. Moreover, 4-D AFM methodology also permits to rinse cells with fresh medium and observe the reversibility of changes made by a certain agent. The proof-of-concept example using cytochalasin B as an agent was shown recently (Zapotoczny et al. 2019b). We presented that cytochalasin B effect is reversible in ~ 1 h.

Over the years, a number of fenestrae-associated cytoskeletal structures were described using EM. For example, each fenestra was reported to be surrounded by a FACR. It was reported that new fenestrae are formed from FFC, flat spots in the central part of a sieve plate with fenestrae radiating from it in tornado-like rows. DFC were reported in LSECs apparently loosing fenestrae, suggesting an opposite process. However, our full understanding on the dynamic nature of these peculiar structures remains largely unknown. With the advent of novel AFM methodology, we could better define those intriguing structures. Our recent experimentation so far showed that fenestrae can be formed from FFCs but also without FFCs. We also collected indications that FFCs can be formed without cytoskeletal-altering drugs, implying their natural occurrence in non-stimulated LSECs—although at a lower frequency. Moreover, DFC could also be observed in vitro via 4-D AFM. Tracking of fenestrae in live LSECs allowed identifying a completely new phenomenon, namely that fenestrae can close and re-open. The process of maintaining open fenestra is dependent on the actin-spectrin cytoskeleton. In a recent report, we showed that destabilization of spectrin-actin cytoskeleton forming FACR leads to rapid closing of fenestrae and also can cause fenestrae “blinking”, i.e. a rapid switching between open and close state of fenestra (Zapotoczny et al. 2019a). Those processes can only be analysed using live cell imaging techniques.

### Comparative measurements using different microscopic techniques

Due to methodological limitations, AFM cannot be adapted for in vivo measurements. Until today, there are no reports on the dynamics of fenestrae in vivo. So far, we are limited to 4-D in vitro studies of LSEC fenestrae. Nonetheless, we believe that 4-D AFM allowed for a breakthrough in understanding of the processes driving fenestrae in LSECs. The observations about LSECs changing their porosity (processes of closing, maintenance, opening of fenestrae) and the involvement of different cytoskeletal elements are crucial for planning future experimental setups and therapeutic possibilities. It is important to note that AFM imaging allows for observing changes in cell morphology, including topography and mechanical mapping, but it can also be used for the identification of superficial biomolecules. Alsteens et al. elegantly showed that using sinusoidal mode of FD curves acquisition, the resolution of a few nanometres was feasible on bacterial surface (Alsteens et al. 2013). Nevertheless, the identification of cytoskeletal components which are located beneath the cell membrane of intact cells remains impossible with AFM. To achieve more complex information about the processes driving fenestrae, we need comparative imaging using different microscopy techniques. Recently, it has been shown for the first time that the coupling of AFM with super-resolution nanoscopy, namely, dSTROM, is a possibility. We tested our hypothesis about actin-spectrin cytoskeleton responsibility in regulation of fenestrae openings using complementary information from both microscopy techniques.

Correlative SEM and AFM studies have been briefly reported (Braet et al. 2018). In this study, a marking point on a sample was used to obtain images of the same area studied using these two microscopy technologies. The experiment allowed to assess the differences between the dimensions of fenestrae. The same fenestrae observed in AFM were of about 4.6 ± 2.3% smaller than those observed using SEM. The difference is probably caused by tip geometry as AFM images always present convoluted information of both sample and tip shapes.

## 4-D atomic force spectroscopy: elasticity as a parameter describing LSECs physiological state

**Fig. 5:**
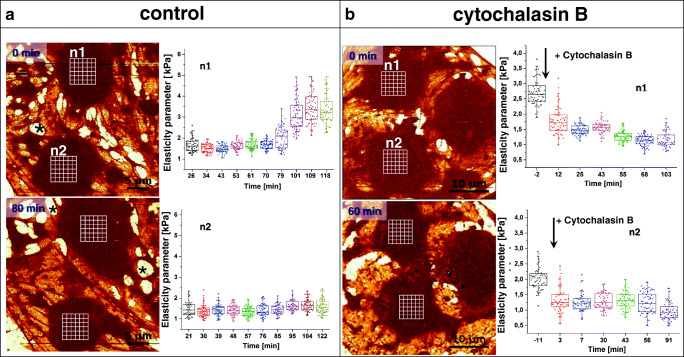
Two experiments using 4-D AFM/AFS are presented. Left panel presents control experiments in which cells were investigated for prolonged time. Right panel presents experiment in which cells were treated with cytochalasin B. Control: Images represent the stiffness of cells at the beginning of the experiment and after 80 min. Between the acquisitions of the images AFS was performed in the area corresponding to the top of the nuclei. We observe a correlation between stiffening of cell (increased elasticity parameter) and appearance of gaps (asterisks). Cytochalasin B: Similar experiment as the control one, but in time 0 cells were treated with 21 μM cytochalasin B. Depolymerization of actin resulted in both increase in fenestrae number and decrease in cell elasticity parameter. Grid of 8 × 8 FD curves was set over 6 × 6 μm^2^ area above the top of cell nucleus

Fenestrae and sieve plates are widely accepted structural features of LSECs. AFM-based indentation spectroscopy offers yet another option of probing the cell mechanical properties at the nanoscale (for a review, see (Szymonski et al. 2015)). Changes in nanomechanics have often been used as a marker of pathology. For example, the difference between stiffness of normal and cancerous cells was reported to be one order of magnitude (Lekka et al. 1999). Stiffening of the tissue was also used as a parameter describing progression of liver steatosis (Mueller and Sandrin 2010). Elasticity was reported as a marker allowing characterization of early stages of endothelial dysfunction (Szymonski et al. 2015), and the actin cytoskeleton was found to be the main component that determines the elasticity of cells (Grady et al. 2016), especially at small strains (Kubitschke et al. 2017). Therefore, we pose the question whether elasticity (as evaluated by determination of Young’s modulus) can be validated as a new biophysical indicator and/or additional structural parameter to describe the structural state of LSECs. While both the number of fenestrae and the elasticity depend on the actin cytoskeleton, we pursued this hypothesis knowing that one of these parameters could predict the other. In the example given in Fig. 5, we present a low-magnification AFM image of two LSECs in vitro and their corresponding Young’s modulus values in time progression.

After the first image was acquired (defined as 0 min), the elasticity was further evaluated using atomic force spectroscopy (AFS). Namely, we selected an area over the cell nucleus (n1) and acquired several FD curves (each small white square corresponds to individual FD curve) to determine the Young’s modulus. Next, we selected the second cell (*n2*) and followed FD curves acquisition. We followed performing AFS of both cells interchangeably. After an hour of force mapping (80 min from the start of the initial image), another image was recorded (defined as 80 min). The difference between the images clearly illustrates that one of the cells (*n1*) formed many gaps (Fig. 5, asterisks), and as a result, most of the fenestrae in this subcellular part of the cell disappeared. The second cell (n2) maintained the typical fenestrated morphology. Continuously collecting AFS maps after the acquisition of the second image indicated a significant stiffening (increased Young’s modulus) of one of the cells (n1), while the elasticity parameter of the second cell remained constant in this example. In our hands, formation of gaps and the stiffening of cells were correlated with loosing fenestrae. A similar experiment was done using cytochalasin B-treated LSECs (Fig. 1, bottom panel). The family of cytochalasins are actin-altering drugs, which typically result in actin depolymerization. LSECs treated with cytochalasins respond with a gradual increase in porosity up to 300% of the initial baseline value (Steffan et al. 1987; Braet et al. 1996a; Zapotoczny et al. 2017c). Moreover, it was demonstrated for variety of cell types; the depolymerization of actin due to the treatment with cytochalasins caused significant softening of the cell cortex (Pogoda et al. 2012; Grimm et al. 2014; Grady et al. 2016). Here, we observed a similar response of LSECs. Using 4-D AFM, we could determine that the most prominent decrease in elasticity parameter was observed in the first 5 min after cytochalasin B treatment (in comparison to the 15 min of the most prominent increase in porosity in Fig. 4).

AFS can be applied to whole cells in a so-called force volume mode, allowing to reconstruct images of elasticity distribution in cells (Radmacher 2007; Zapotoczny et al. 2017a). However, such imaging is time-consuming thereby limiting its application for high-throughput purposes. In QI mode, similar to force volume mode, a full FD curve is recorded in each given single pixel. By employing such AFM workflow, five LSECs were randomly selected, and their morphologies and nanomechanics were traced in time for 5 h using QI (Fig. 5 & Supplementary animation 3). To calculate Young’s modulus, we applied the Hertz-Sneddon mechanical model (Sneddon 1965) assuming the conical shape of the probing tip.


ESM 3(MP4 9501 kb)


**Fig. 6:**
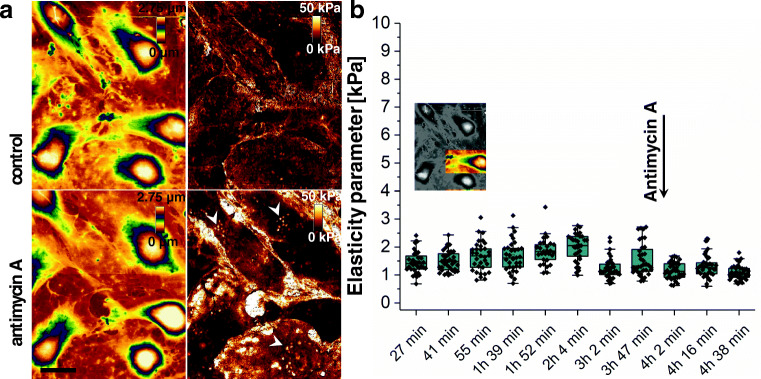
4-D AFM images of the group of several live LSECs. (a) Left pictures represent the topography images and right pictures calculated Young’s modulus. Upper row represents selected image corresponding to control LSECs. Five hours animation of changes in LSECs topography and elasticity is presented as Supplementary animation 3. Bottom image represents the same LSECs treated with 1.9 μM antimycin A for 1 h. Lipid droplets growth in number and size and can be visible in images of Young’s modulus (arrowheads). Scale bar 10 μm. z-scale was set the same for all images. Image size and resolution: 45 μm × 55 μm, 300 × 367 pixels. (b) Between the acquisitions of images presented in Supplementary animation 3, force volume mode was applied to calculate Young’s modulus of individual cells in the nuclear zone (see, Fig. 5) and the time variations in Young’s modulus of one representative cell was presented. Addition of 1.9 μM antimycin A was indicated. Grid of 6 × 6 FD curves was set over 5 × 5 μm^2^ area above the top of cell nucleus

At first, LSECs were measured for 3 h in the cell culture medium to show that only minor changes in the elasticity occurred and when no chemical stimulation is applied. The migration of cells remains random and does not depend on the direction of scanning. In another report, it was shown that continuous scanning in contact mode AFM resulted in an endothelial cell’s remodelling and orientation in the scanning direction (Targosz-Korecka et al. 2016). In QI mode, the contact time between the tip and the cell is vastly reduced and the loading force 2–3 times smaller than in the mentioned report. Although we could not see any changes induced by the tip, we cannot exclude that some remodelling of the LSEC cytoskeleton resulted as a response to the poking with AFM tip during QI measurements. After acquisition of each image, the elasticity for each cell was assessed using AFS (see, Fig. 5).After 3 h, LSECs were treated with 1.9 μM antimycin A. This drug is a well-described inhibitor of mitochondrial energy production. The concentration of the drug was previously reported to be sufficient to induce closing of fenestrae in 1 h (Fig. 4)(Braet et al. 2003; Zapotoczny et al. 2017c). After 1 h of antimycin A treatment, we observed an overall stiffening of cells (i.e. corresponding to brighter contrast in Fig. 6, antimycin A). Similar to another report where Raman spectroscopy was used to investigate LSECs (Szafraniec et al. 2019), we distinguished numerous lipid droplets around cell nuclei (Fig. 6, arrowheads). Their presence was confirmed using Oil Red O staining. The role of formation of lipid droplets in cells is plentiful, but direct processes regulating their formation remain elusive (Koizume and Miyagi 2016; Welte and Gould 2017). It has been reported that the redox imbalance results in cellular stress, which in turn activates lipogenesis and induce lipid droplets formation (Lee et al. 2015; Koizume and Miyagi 2016). Similar to these reports, we observed that the number and size of lipid droplets increased after antimycin A treatment. The most pronounced changes in elasticity of LSECs treated with antimycin A were observed on the cell peripheries (i.e. cell cytoplasm) in contrast to only minor changes in the nuclear zone. We, therefore, highlight the potential of the approach when simultaneous observation of LSECs morphology and elasticity modulus is performed using QI. Such approach provides complex information about the distribution of changes in values of markers allowing assessing the pathology of cells (porosity, elasticity, lipid droplets). We emphasize that the absolute values of Young’s modulus calculated using QI in the areas over cell nuclei were more than twice time larger than those collected when applying traditional AFS. The speed of the individual FD curve collection in these experiments was 50 times larger (100 μm/s for quantitative imaging, compared to 2 μm/s in traditional AFS). Similar difference was shown for variety of cell types (Lekka et al. 2012a, b; Nawaz et al. 2012) and explained as viscoelastic response of cells (Li et al. 2008; Nawaz et al. 2012). It indicates that 4-D AFM (or 4-D AFS, while we use QI matrix for calculation of Young’s modulus in each point) is useful when tracking of alterations in individual cells is required. To assess the elasticity of cells more accurately, a classical approach to the force mapping is required. Interestingly, we could not see the significant change in LSEC elasticity when force volume mode was performed (Fig. 6b), while observed changes in elasticity occurred mainly in the perinuclear zone. In a recent report, it was demonstrated that only detailed analysis of each FD curve, using multi-layered system approximation, revealed the differences in the elasticity of LSECs isolated from mice on control and high fat diet (Rusaczonek et al. 2019). However, such approach is time-consuming and requires analysis of each FD curve independently.

## Conclusion and future outlook

Recent advances in AFM microscopy provided new insights into the dynamic processes of fenestrae formation, migration, disappearance and closing. After 50 years of describing of LSEC-fenestrae under chemical-fixed conditions, it was only until recently that we were able to collect insights on fenestrae at high resolution in living LSECs over time. Several fenestrae-associated structures that were described before using chemically processed specimens were confirmed and monitored in living cells using the latest advances in 4-D AFM. Furthermore, we disclosed additional aspects on the turn-over, movement and biophysical properties of (grouped) fenestrae. We revisited that fenestrae are rather short-lived structures and that they can readily change their number, shape and size during their ‘short’ lifespan. And those changes go hand-in-hand with changes in elastic properties of LSECs.

We foresee that future experiments will further focus on the processes of fenestrae formation versus loss via newly (to be discovered) fenestrae-altering agents. AFM could also shed a light into the molecular composition of fenestrae which drive these processes. The use of functionalized AFM tips seems to be the next logic step. Furthermore, high-throughput 4-D AFM holds big promise as hundreds of LSECs could be readily scanned allowing the quantification of ten thousands of fenestrae in one single assay. Finally, correlative imaging techniques can open up an entire new direction to map the biophysical and structural properties of LSECs. 4-D AFM combined with optical nanoscopy for example could become a standardized approach for researching the influence of physical and chemical stimuli on LSEC fenestrae and associated protein structures.
